# Dimethyl Trisulfide Diminishes Traumatic Neuropathic Pain Acting on TRPA1 Receptors in Mice

**DOI:** 10.3390/ijms22073363

**Published:** 2021-03-25

**Authors:** Ágnes Dombi, Csenge Sánta, István Z. Bátai, Viktória Kormos, Angéla Kecskés, Valéria Tékus, Krisztina Pohóczky, Kata Bölcskei, Erika Pintér, Gábor Pozsgai

**Affiliations:** 1 Department of Pharmacology and Pharmacotherapy, Medical School, University of Pécs, Szigeti u. 12, H-7624 Pécs, Hungary; agnes.borzsonyi@aok.pte.hu (Á.D.); scsenge98@gmail.com (C.S.); istvanzbatai@gmail.com (I.Z.B.); viktoria.kormos@aok.pte.hu (V.K.); angela.kecskes@aok.pte.hu (A.K.); valeria.tekus@aok.pte.hu (V.T.); pohoczkykriszti@gmail.com (K.P.); kata.bolcskei@aok.pte.hu (K.B.); erika.pinter@aok.pte.hu (E.P.); 2 Molecular Pharmacology Research Group, Szentágothai Research Center, University of Pécs, Ifjúság útja 20, H-7624 Pécs, Hungary; 3 Department of Pharmacology, Faculty of Pharmacy, University of Pécs, Rókus u. 2, H-7624 Pécs, Hungary

**Keywords:** TRPA1, somatostatin, SST_4_, dimethyl trisulfide, neuropathic pain, partial ligation, sciatic nerve, RNAscope, microglia

## Abstract

Pharmacotherapy of neuropathic pain is still challenging. Our earlier work indicated an analgesic effect of dimethyl trisulfide (DMTS), which was mediated by somatostatin released from nociceptor nerve endings acting on SST_4_ receptors. Somatostatin release occurred due to TRPA1 ion channel activation. In the present study, we investigated the effect of DMTS in neuropathic pain evoked by partial ligation of the sciatic nerve in mice. Expression of the mRNA of *Trpa1* in murine dorsal-root-ganglion neurons was detected by RNAscope. Involvement of TRPA1 ion channels and SST_4_ receptors was tested with gene-deleted animals. Macrophage activity at the site of the nerve lesion was determined by lucigenin bioluminescence. Density and activation of microglia in the spinal cord dorsal horn was verified by immunohistochemistry and image analysis. *Trpa1* mRNA is expressed in peptidergic and non-peptidergic neurons in the dorsal root ganglion. DMTS ameliorated neuropathic pain in *Trpa1* and *Sstr4* WT mice, but not in KO ones. DMTS had no effect on macrophage activity around the damaged nerve. Microglial density in the dorsal horn was reduced by DMTS independently from TRPA1. No effect on microglial activation was detected. DMTS might offer a novel therapeutic opportunity in the complementary treatment of neuropathic pain.

## 1. Introduction

According to the definition of the International Association for the Study of Pain (IASP), neuropathic pain arises due to injury or disease of the somatosensory nervous system [1]. An increasingly common cause of peripheral nerve injury is diabetes. An estimated 28% of diabetic patients in the US develop peripheral neuropathy. Besides painful symptoms, this condition might lead to foot ulceration, amputation and conflict with everyday activities [2]. The overall prevalence of neuropathic pain in the European patient population is estimated to be 7% and is increasing [3]. Mostly based on clinical experience, plentiful drugs are utilized in the pharmacotherapy of neuropathic pain. Non-invasive neurostimulation and invasive interventions are emerging therapeutic tools, too. According to epidemiological data, despite the broad range of medicinal options, many patients fail to access proper treatment [4]. The number of patients responding to placebo is especially large. Typically, only 10–25% more patients benefit from pharmacotherapy than from placebo application [5]. To worsen the situation, mainline gabapentinoid drugs of neuropathic pain are emerging substances of abuse [6,7]. These circumstances necessitate further research in novel directions for pharmacological treatment and target mechanisms in the field of neuropathic pain.

Transient receptor potential ankyrin 1 (TRPA1) ion channels are non-selective cation channels belonging to the transient receptor potential family. They are mostly expressed on primary nociceptor nerve endings, central nervous system neurons, glial structures and inflammatory cells [8,9,10]. TRPA1 channels are polymodal and can be activated by endogenous products of oxidative and nitrergic stress, i.e., products of lipid peroxidation, nitrated lipids, H_2_O_2_ and polysulfides [11]. The involvement of TRPA1 channels in neuropathic pain is illustrated by their upregulation in primary nociceptors in animal models of the condition, as well as by mitigation of neuropathic pain by antagonism and genetic deletion of *Trpa1* [8,12,13].

Macrophages and Schwann cells might play an important role in the pathomechanism of mechanical allodynia accompanying neuropathic pain. In the case of traumatic nerve damage, chemokines are released from the damaged nerve. Chemokines recruit macrophages to the site of nerve damage. Macrophages, through an interplay with Schwann cells, contribute to the activation of TRPA1 channels of nociceptors. TRPA1 activation induces mechanical allodynia. This effect relies on the expression of TRPA1 in both macrophages and Schwann cells [8,14].

TRPA1 ion channels can be activated by polysulfides. Inorganic polysulfides might arise in cells from reactions of sulfide with nitric oxide, reaction of sulfide with hypochlorous acid or produced by the enzyme 3-mercaptopyruvate sulfurtransferase [15,16,17]. Dimethyl trisulfide (DMTS) is an organic dialkyl polysulfide. It is present in garlic, the common earwig and fermented food [18,19,20]. Unlike sulfide, polysulfides are potent agonists of TRPA1. Both sodium polysulfide (POLY) and DMTS induce calcium signals in primary cultures of sensory neurons expressing TRPA1, as well as in cell lines transfected with the ion channel. Similar data could be produced by a patch clamp [16,21,22]. While POLY is unstable and short-lived, DMTS is a stable chemical. DMTS has an elimination half-life of 35 min in rodents combined with other advantageous pharmacokinetic properties [23]. Previously, we investigated the effect of POLY and DMTS in injury- or inflammation-evoked pain. POLY was tested in carrageenan-induced paw inflammation and DMTS was studied in paw inflammation and pain due to heat trauma. Both POLY and DMTS attenuated mechanical allodynia, but only DMTS blunted vascular and cellular inflammatory parameters, demonstrated by reduced paw swelling and myeloperoxidase-driven luminescence [20,22].

The antinociceptive effect of TRPA1 activation is in contrast with the efficacy of TRPA1 antagonists in models of neuropathic pain and other painful conditions. TRPA1 expression in primary sensory neurons releases neuropeptides from terminals upon calcium influx due to channel opening. Some of them (e.g., substance P, calcitonin gene-related peptide) contribute to neurogenic inflammation by prompting vasodilatation and plasma leakage, but others, like somatostatin, have antinociceptive and anti-inflammatory effects. The cyclic peptide somatostatin is an endocrine mediator that reaches its remote targets by the systemic circulation. The pain-relieving effect was found to rely on somatostatin SST_4_ receptors. Genetic deletion of the *Sstr4* receptor diminished the action of somatostatin in various animal models of pain. On the other hand, receptor agonists could reproduce the beneficial effect [24]. In our previous experiments, the antinociceptive action of POLY and DMTS was transmitted by SST_4_, and demonstrated by its absence in receptor gene knockout mice. However, amelioration of vascular and cellular inflammation by DMTS was independent of SST_4_ [20,22]. Messenger RNA of *Sstr4* somatostatin receptors was identified in the spinothalamic neurons of the dorsal horn of the spinal cord by the RNAscope method, marking a putative site of action of DMTS-released somatostatin [25].

Based on our preceding results, in the present study we embarked to explore the effect of DMTS in a partial nerve ligation-induced traumatic neuropathy model. Expression of *Trpa1* mRNA in dorsal root ganglia was detected by RNAscope. Involvement of TRPA1 ion channels and SST_4_ somatostatin receptors was tested with knockout mice. Neuropathic pain was characterized by mechanical allodynia of hind paws, immunohistochemistry of lumbar spinal cord, as well as luminescent imaging of radicals released from macrophages at the site of the nerve injury.

## 2. Results

### 2.1. Trpa1 mRNA Is Expressed in Dorsal Root Ganglion Neurons

**Figure 1:**
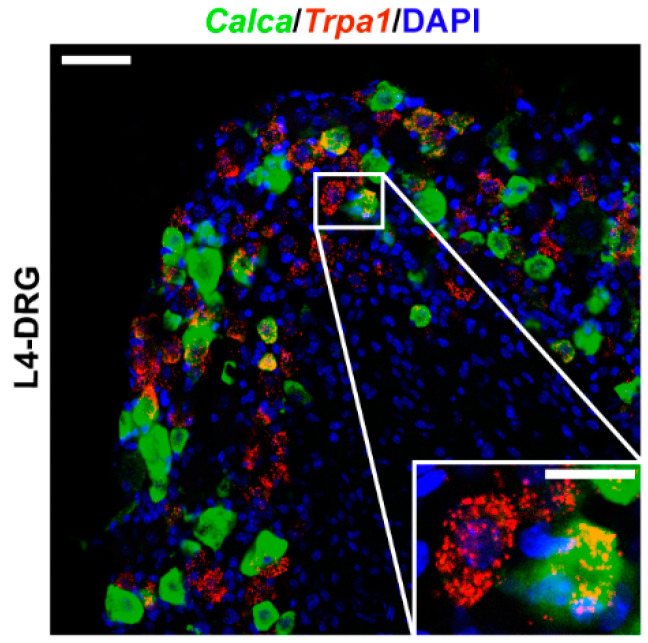
Confocal image of *Trpa1* mRNA expression on dorsal root ganglion (DRG). *Trpa1* (red) and *Calca* (CGRP, green) mRNA expression counterstained with DAPI are shown in mouse L4-DRG. Scale bar: 50 µm, inset scale bar: 20 µm.

Mouse *Trpa1* mRNA was detected in L4-dorsal root ganglion (DRG) using multiplex fluorescent RNAscope in situ hybridization. In DRG, *Trpa1* was localized on calcitonin gene-related peptide (CGRP)-positive sensory neurons. However, co-localization of *Trpa1* with *Calca* (encoding CGRP) was only partial, not all *Trpa1*-expressing neurons were peptidergic, also not all peptidergic sensory neurons were *Trpa1* positive (Figure 1).

### 2.2. Dimethyl Trisulfide Alleviates Neuropathic Pain

**Figure 2:**
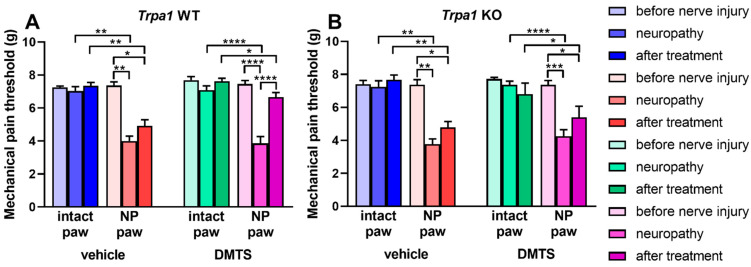
DMTS alleviates mechanical hyperalgesia in the hind paws *Trpa1* WT animals. Mechanical pain threshold of the hind paws of *Trpa1* WT (**A**) and KO (**B**) animals with unilateral sciatic nerve lesion before surgery, after neuropathy has developed and after vehicle or DMTS treatment. *n* = 6–8; one-way analysis of variance (ANOVA) followed by Tukey’s tests; * *p* < 0.05, ** *p* < 0.01, *** *p* < 0.001, **** *p* < 0.0001 vs. indicated groups. NP: neuropathic side (right).

Neither DMTS nor respective vehicle treatment had any effect on the mechanical pain threshold of uninjured hind legs. Presence or absence of functional TRPA1 ion channels did not influence mechanical sensitivity of the intact paws. Insertion of a suture into the sciatic nerve caused a marked drop of the mechanical pain threshold after 7 days when compared to the values detected before the surgery. This condition was an inclusion criterion for the animals. The vehicle of DMTS left the lowered pain threshold unrelieved (Figure 2A,B). Application of DMTS restored mechanical sensitivity of the operated hind paw to the normal level in *Trpa1* WT animals, indicated by a statistical difference between threshold values detected after surgery, but before DMTS administration and those taken after treatment (Figure 2A, *n* = 7–8). No amending effect of DMTS occurred in mice genetically lacking functional TRPA1 channels (Figure 2B). These findings imply that TRPA1 is a crucial target molecule of the effect of DMTS in neuropathic pain.

### 2.3. Beside TRPA1 Ion Channel, SST_4_ Somatostatin Receptor Mediates Antihyperalgesic Effect of DMTS

**Figure 3:**
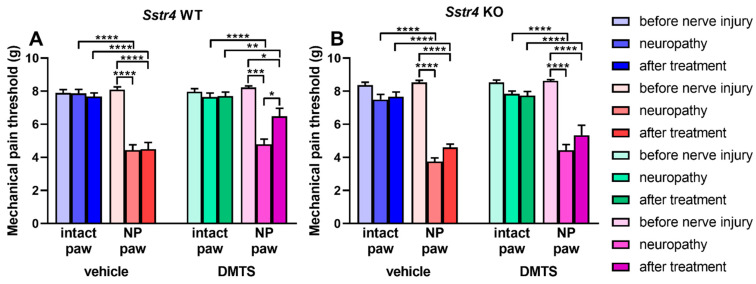
DMTS reduces mechanical hyperalgesia in the hind paws *Sstr4* receptor WT animals. Mechanical pain threshold of the hind paws of *Sstr4* WT (**A**) and KO (**B**) animals with unilateral sciatic nerve lesion before surgery, after neuropathy has developed and after vehicle or DMTS treatment. *n* = 6–7; one-way ANOVA followed by Tukey’s test; * *p* < 0.05, ** *p* < 0.01, *** *p* < 0.001, **** *p* < 0.0001 vs. indicated groups. NP: neuropathic side (right).

In agreement with the results in *Trpa1* WT and KO mice, DMTS or vehicle administration did not alter mechanical threshold in hind legs of *Sstr4* WT and KO animals with unhurt sciatic nerves. Vehicle of DMTS did not affect mechanical sensitivity of intact paws or ones with damaged sciatic nerves. Surgical trauma of the sciatic nerve according to Seltzer curtailed pain threshold after 7 days in *Sstr4* WT and KO animals (Figure 3A,B). Seven doses of DMTS increased the mechanical pain threshold in *Sstr4* WT animals indicated by statistical difference between the sensitivity values of neuropathic hind legs obtained before and after DMTS treatment (Figure 3A, *n* = 6–7). In mice genetically lacking *Sstr4*, DMTS did not exhibit any antihyperalgesic effect (Figure 3B). These findings denote that beside TRPA1, SST_4_ is another major player in the ameliorating effect of DMTS in traumatic mononeuropathy.

### 2.4. Dimethyl Trisulfide Does Not Alter Macrophage Activity Around the Damaged Sciatic Nerve

**Figure 4:**
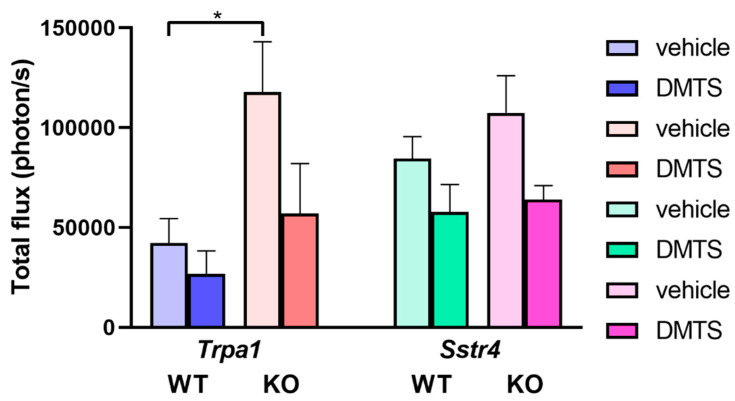
DMTS treatment does not inhibit macrophage cell activity at the site of the sciatic nerve lesion. Lucigenin luminescence expressed as total light flux (photons/s). Data were collected in *Trpa1* WT and KO, as well as in *Sstr4* receptor WT and KO mice at the site of unilateral surgical sciatic nerve lesion. *n* = 6–9; Kruskal–Wallis test followed by Dunn’s test; * *p* < 0.05 vs. indicated group.

Chemokines and radicals released from activated macrophages were found to contribute to the pathomechanism of neuropathic pain [8,14]. We commenced detection of NADPH oxidase activity of macrophages around the sutured sciatic nerves, illustrated by lucigenin bioluminescence. Data collected from the legs with a nerve injury are plotted only because intact legs do not evince any radiation. DMTS displayed a trend of reduced luminescence in *Trpa1* WT and KO animals compared to its vehicle. Macrophage activity was significantly larger in vehicle-treated *Trpa1* KO mice than in WT ones. Similar observations can be made in *Sstr4* mice, except that no difference manifests between WT and KO animals (Figure 4).

### 2.5. DMTS Mitigates Microglia Density in the Spinal Cord Dorsal Horn Independently from TRPA1

**Figure 5:**
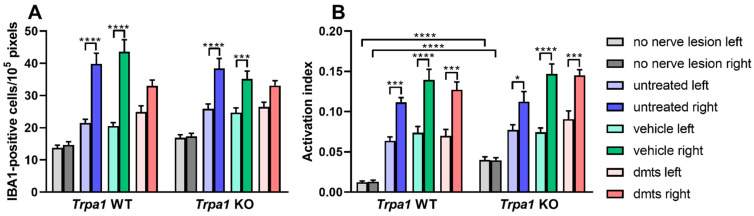
TRPA1-dependent relief of traumatic mononeuropathy by DMTS is not mediated by IBA1-positive cells of the spinal cord dorsal horn. Number (**A**) and activation index (**B**) of IBA1-positive microglia in the spinal cord dorsal horn of *Trpa1* WT and KO mice with unilateral surgical lesion of the sciatic nerve. An activation index closer to 1 indicates larger cell activation. *n* = 3–5 animals, 2–13 slides per animal. We evaluated 13–34 photographs per group for cell density and 11–35 photographs to calculate activation indices. One-way ANOVA followed by Dunnett’s test. * *p* < 0.05, *** *p* < 0.001, **** *p* < 0.0001 vs. indicated groups.

**Figure 6:**
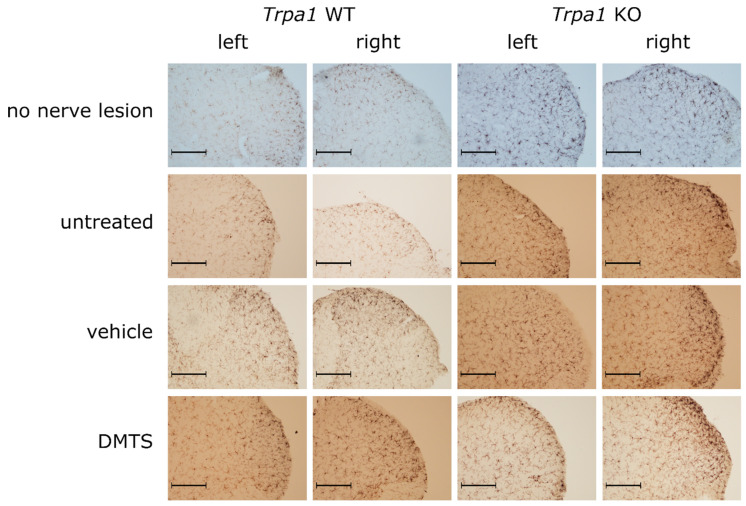
Representative micrographs used for the calculation of the density of IBA1-positive microglia in the spinal cord dorsal horn. Immunohistochemistry was visualized with 3,3′-diaminobenzidine. Images were taken with a 10× objective. Size bars indicate 200 µm.

The density of ionized-calcium-binding-adaptor-molecule-1-positive (IBA1) cells was investigated in laminae 1 and 2 of the dorsal horn in the spinal cord. The density was higher in animals with a sciatic nerve lesion irrespective of treatment group and genotype compared to mice with no nerve lesion. Interestingly, samples contralateral to the lesion showed significantly increased microglia density, compared to the not-operated control, too. A marked increase of density values on the lesion side (right) were detected compared to the contralateral side in mice receiving no treatment or vehicle of DMTS. No such difference was detected in either *Trpa1* WT or KO not operated animals and in ones with nerve lesion plus DMTS treatment. Lack of elevated microglia density at the lesion side in the DMTS group indicates protective effect of DMTS. Presence of this effect in *Trpa1* WT and KO mice demonstrates that it is not mediated by TRPA1 activation (Figure 5A and Figure 6).

**Figure 7:**
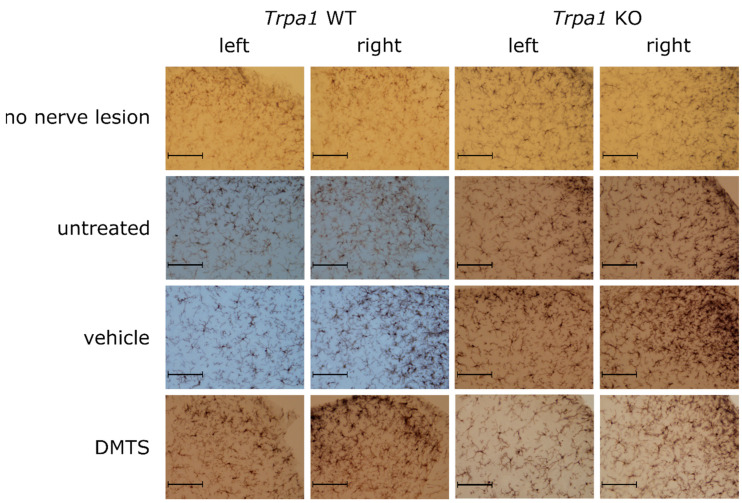
Representative micrographs used for the detection of the activation index of IBA1-positive cells in the spinal cord dorsal horn. Immunohistochemistry was visualized with 3,3′-diaminobenzidine. Images were taken with a 20× objective. Size bars equal to 100 µm.

*Trpa1* WT and KO mice with sciatic nerve lesion exhibited increased microglia activation compared to those with no nerve damage on both the lesion and the contralateral sides. Surprisingly, intact *Trpa1* KO animals without nerve lesion exhibited larger microglia activation on both left and right sides than their WT counterparts. Mice with damaged sciatic nerves displayed greater microglia activation on the ipsilateral side than on the contralateral side in untreated, vehicle and DMTS groups (Figure 5B and Figure 7).

## 3. Discussion

Our study reveals that DMTS effectively palliates mechanical hyperalgesia induced by traumatic injury of the sciatic nerve. Data collected with the help of mice genetically lacking TRPA1 ion channels or SST_4_ receptors identify the channel and the receptor as pivotal conveyors of the anti-neuropathic effect of DMTS. RNA expression of *Trpa1* in DRG neurons and that of *Sstr4* in spinothalamic neurons make the latter a potential target of DMTS-released somatostatin. Luminescent imaging of macrophage-derived NADPH oxidase around the nerve lesion elucidated that DMTS does not inhibit inflammation and oxidative stress elicited by peripheral macrophages. Immunohistological quantification of microglia in the spinal cord dorsal horn rules out microglia as targets of the antinociceptive effect of DMTS.

TRPA1 expression was detected in macrophages [26,27]. TRPA1 was also reported to regulate polarization of macrophages towards M1 or M2 phenotypes [28]. M1 or classically activated macrophages promote tissue damage, while M2 or alternatively activated macrophages contribute to repair mechanisms, among other functions. TRPA1 activation promoted the alternative activation pathway that might be beneficial in the repair of nerve damage and prevention of neuropathic pain. Microglia in the dorsal horn of the spinal cord were identified to participate in the development of neuropathic pain after partial ligation of the sciatic nerve, too [29,30]. The density of IBA1-positive microglia was elevated on the lesion side in laminae 1 and 2 in the dorsal horn of the spinal cord in mice [31]. Our data showed an increased density of ipsilateral microglia in the spinal cord dorsal horn. DMTS reduced the number of ipsilateral microglia in the dorsal horn and had a similar, but statistically insignificant effect on macrophages at the site of nerve lesion. However, these effects do not mediate TRPA1-dependent antineuropathic effect of DMTS, as they occur in KO mice.

One limitation of our study is that our data do not fully coincide with data in the literature regarding effects of organic and inorganic polysulfides on macrophages and microglia. The effect of POLY was investigated in the RAW264.7 murine macrophage cell line. POLY inhibited the release of inflammatory cytokines, transcription factor nuclear factor kappa-light-chain-enhancer of activated B cells (NF-κB) and Toll-like receptor 4 (TLR4) signaling [32]. POLY mitigated the activity of Ca^2+^/calmodulin-dependent protein kinase II (CaMKII) in the same macrophage cell line. CaMKII contributes to the formation of M2 tumor-associated macrophages and necrosis of atherosclerotic plaques [33,34,35]. There are not many papers available on the effects of DMTS. However, the effects of a similar organic polysulfide—diallyl trisulfide (DATS)—have been studied in more detail. DATS exerted similar effects in LPS-stimulated RAW264.7 cells to those of POLY with respect to inflammatory cytokine secretion, NF-κB and TLR4 signaling [36]. In our experiments, DMTS failed to reduce the activity of macrophages at the site of the sciatic nerve lesion in a statistically significant manner. The murine BV-2 cell line was used to test the effect of POLY and DATS in microglia. Nitroxyl, a product of POLY, mitigated LPS-induced apoptosis in BV-2 cells [37]. DATS exhibited an effect similar to that of POLY in BV-2 cells [38]. These findings are partially in line with ours: DMTS curtailed microglial density in the dorsal horn, albeit histological markers of microglial activation were not affected. This incongruence might be explained by the promiscuity of polysulfides. Our previous work focused on the interaction of POLY and organic DMTS with the TRPA1 ion channel. We described activation of TRPA1 by both substances. We used CHO cells, as well as primary cultures of murine trigeminal ganglion neurons. TRPA1 activation was detected by calcium-sensitive fluorescent markers (either in a ratiometric way or not) and by automated patch clamp. Ion channel activation could be inhibited by selective TRPA1 antagonist HC030031 and lack of TRPA1 [16,22]. However, polysulfides react potentially with any thiol groups of proteins and change the protein function. A multitude of proteins have been reported to be modulated by polysulfides, including actin, glyceraldehyde-3-phosphate dehydrogenase (GAPDH), nuclear factor κB (NF-κB), ATP-sensitive potassium channel (KATP), protein tyrosine phosphatase 1B (PTP1B), Kelch-like ECH-associated protein-1 (Keap1) and phosphatase and tensin homolog (PTEN) [39,40]. Divergent actions of DMTS on numerous proteins at different levels of the organism might evoke opposing effects in macrophages.

DMTS appears to be a much more promising candidate in the therapy of neuropathic pain than POLY. Inorganic sodium polysulfide possesses immense reactivity. The reaction kinetics of POLY and glutathione have recently been outlined by resonance synchronous spectroscopy [41]. The half-life of POLY was found to be 78 s. In our study POLY was administered intraperitoneally. Substances absorbed from the peritoneum are subjected to presystemic elimination [42]. Absorption kinetics of the intraperitoneal route are different from those of the intravenous one. They rather resemble oral absorption. Based on the relatively slow absorption and extensive first pass metabolism by glutathione in hepatocytes, it is fair to assume that only a minor part of the POLY dose might reach the central nervous system or even peripheral nociceptors expressing TRPA1. On the other hand, DMTS possesses an elimination half-life of 36 min in the rat and readily crosses the murine blood–brain barrier [23,43]. DMTS molecules have much better chance to reach and activate peripheral or central TRPA1 channels. These findings seem to conflict with our earlier data demonstrating efficacy of POLY in carrageenan-induced paw inflammation, as well as that of polysulfide originating from slow-release sulfide donor GYY4137 [16,20]. In these cases, the inflammation driving nociception was still ongoing when POLY was applied. We provided evidence that GYY4137-delivered polysulfide is formed at the site of cellular inflammation, providing better availability at the target location.

Our results specify that the ameliorating effect of DMTs on neuropathic pain is mediated by TRPA1 ion channels and SST_4_ somatostatin receptors. According to preceding data, SST_4_ receptor activation is most probably downstream to the opening of TRPA1 channels. TRPA1 channels are known to be co-expressed in primary nociceptor neurons with TRPV1 ones [44]. Activation of TRPV1 channels with capsaicin lead to somatostatin release into the bloodstream that could be detected by radioimmunoassay in rats [45]. POLY, a TRPA1 agonist, was established to release somatostatin from nerve endings of isolated murine skin [16]. Somatostatin SST_4_ receptor agonists relieve neuropathic pain induced by the same model utilized in the present study in both rats and mice [24,46]. A reasonable target of the somatostatin effect are nociceptor dorsal root ganglion neurons. Selective SST_4_ receptor agonist J-2156 reduced calcium signals in dorsal root ganglion neurons activated by capsaicin [47]. Messenger RNA of *Sstr4* receptor was demonstrated in L4 dorsal root ganglion neurons of the mouse by RNAscope [25]. The above mechanism is not necessarily limited to the peripheral nervous system. TRPA1 ion channels are expressed in cortical and hippocampal neurons [48,49]. Somatostatin is extensively expressed in cortical interneurons [50]. The mRNA of *Sstr4* receptor was identified very early in the rat brain [51]. The presence of SST_4_ was evinced by immunohistochemistry and Western blot in several brain areas, including the parietal cortex and periaqueductal gray [52,53]. Functional importance of SST_4_ in the central nervous system is illustrated by worse performance of *Sstr4* knockout mice in models of anxiety and depression [54,55]. It has to be pointed out that somatostatin release in response to TRPA1 activation has not yet been detected in the central nervous system. In our hands, DMTS treatment lowered the number of IBA1-positive microglia in both *Trpa1* WT and KO mice. This mitigation of neuroinflammation was not mirrored by behavioral data in KO animals. DMTS and other polysulfides do not only interact with TRPA1 ion channels specifically, but target all proteins with functional cysteine residues. Kumar and coworkers found similar reduction of microglia density in the hippocampus of LPS-treated mice in response to intraperitoneal sulfide administration. Less formation of reactive oxygen species is proposed as mechanism of action of sulfide [56]. PI3K/Akt-dependent activation of the Nrf2 signaling pathway is a putative mechanism of the antioxidant effect in smoking-induced cardiac remodeling in rats [57]. Our data did not exemplify an antioxidant effect of DMTS on macrophages at the site of sciatic nerve injury.

According to our data, microglia cells in the spinal cord dorsal horn of naïve *Trpa1* KO mice show different morphology than those of WT ones and appear to be more activated. This is corroborated by elevated oxygen radical production of macrophages at the site of nerve injury indicated by lucigenin luminescence in vehicle-treated *Trpa1* KO mice compared to their WT counterparts. Unfortunately, no publications are available on arborization or activation of microglia in *Trpa1* KO animals. Data are more abundant on macrophages. Larger macrophage activation was found in *Trpa1* KO mice in angiotensin II-induced kidney injury [58]. Classically activated M1 macrophages prevailed in *Trpa1* KO animals with renal ischemia reperfusion injury [59]. *Trpa1* KO macrophages exhibited more severe foam cell formation in response to oxidized LDL [27]. According to conflicting results, macrophage infiltration was smaller in *Trpa1* KO mice in models of corneal wound healing and atopic dermatitis [60,61].

## 4. Materials and Methods

### 4.1. Animals

Male 4–8 week-old C57Bl6/J and *Trpa1* or *Sstr4* gene knockout mice (KO), as well as their wild-type counterparts (WT) were used in the experiments. Both genetically modified strains are based on C57B1/6J animals [62,63]. The mice were bred in the Laboratory Animal House of the Department of Pharmacology and Pharmacotherapy at the University of Pécs under standard pathogen-free conditions with food pellets and water available freely. Experiments conform to the 1998/XXVIII Act of the Hungarian Parliament on Animal Protection and Consideration Decree of Scientific Procedures of Animal Experiments (243/1998), to the European Communities Council Directive of 2010/63/EU and to the requirements of the International Association for the Study of Pain (IASP). Experiments were approved by the Ethics Committee on Animal Research of University of Pécs (license number BA02/2000-30/2016, approved on 24 October 2016). A total number of 198 mice were used in the experiments with the following distribution: C57Bl6/J 1, *Trpa1* WT 56, *Trpa1* KO 54, *Sstr4* WT 42 and *Sstr4* KO 45. The number of animals in separate experimental groups is indicated in figure legends.

### 4.2. RNAscope In Situ Hybridization on Mouse Dorsal Root Ganglion

Male C57Bl6/J (12 weeks old) animals were deeply anesthetized with an overdose of urethane (2.4 g/kg) and perfused transcardially with 4% paraformaldehyde in Millonig’s phosphate buffer. L4 DRG were dissected and postfixed for 24 h at 4 °C, cryoprotected in 30% sucrose in 10% neutral buffered formalin (Merck KGaA, Darmstadt, Germany) for 24 h at 4 °C and frozen in tissue freezing medium (Leica Biosystems, Wetzlar, Germany) on dry ice. Sections of 20 µm were cut using cryostat (CM1850, Leica Biosystems).

RNAscope assay was performed on 20 µm-thick fixed frozen L4-DRG sections of using RNAscope Multiplex Fluorescent Reagent Kit v2 (Advanced Cell Diagnostics, Newark, CA, USA) according to the manufacturer’s protocol. After tissue pretreatment, sections were hybridized with probes specific to mouse *Trpa1* (Cat No. 400211-C2, ACD) and *Calca* (encoding calcitonin gene-related peptide, CGRP, Cat No. 400211-C2, ACD, USA, Cat No. NPR-0004719). Sections were counterstained with DAPI and mounted with ProLong Glass Antifade Mountant (Thermo Fisher Scientific, Waltham, MA, USA) for confocal imaging. DRGs were imaged by using LSM 710 confocal laser scanning microscope (Carl Zeiss, Jena, Germany) Virtual colors were selected to depict fluorescent signals: blue for DAPI, green for *Calca* mRNA (Cyanine 5) and red for *Trpa1* mRNA (Cyanine 5). Images of the respective channels were superimposed to evaluate the co-localization of fluorescent signals. Contrast and brightness were adjusted with Fiji (1.53c, NIH, USA)

### 4.3. Partial Ligation of the Sciatic Nerve

The method of Seltzer was converted for mice [64]. Animals were anesthetized with ketamine (100 mg/kg) and xylazine (10 mg/kg). Surgery was performed unilaterally on the right hind leg under sterile conditions. The sciatic nerve was exposed under microscope through a 5–6 mm skin incision. After careful separation of 30–50% of the proximal common section of the nerve, the separated part was tightly ligated with 8–0 surgical thread. The skin incision was closed with 5–0 absorbable sutures and treated with povidone iodine [24].

### 4.4. Detection of Mechanical Allodynia

Mechanical pain threshold of the hind paws was measured with dynamic plantar esthesiometer (Ugo Basile, Gemonio, Italy). Mice were acclimatized to the apparatus and baseline measurements were taken on 3 occasions before sciatic nerve ligation. Dynamic plantar esthesiometry was repeated 7 days after the surgery. Animals showing no mechanical allodynia of the hind paw on the operated side were excluded from the experiment. Mice were then treated with DMTS or vehicle. Esthesiometry was repeated in 5 hours to test the effect of the treatment.

### 4.5. Dimethyl Trisulfide Treatment

**Figure 8:**
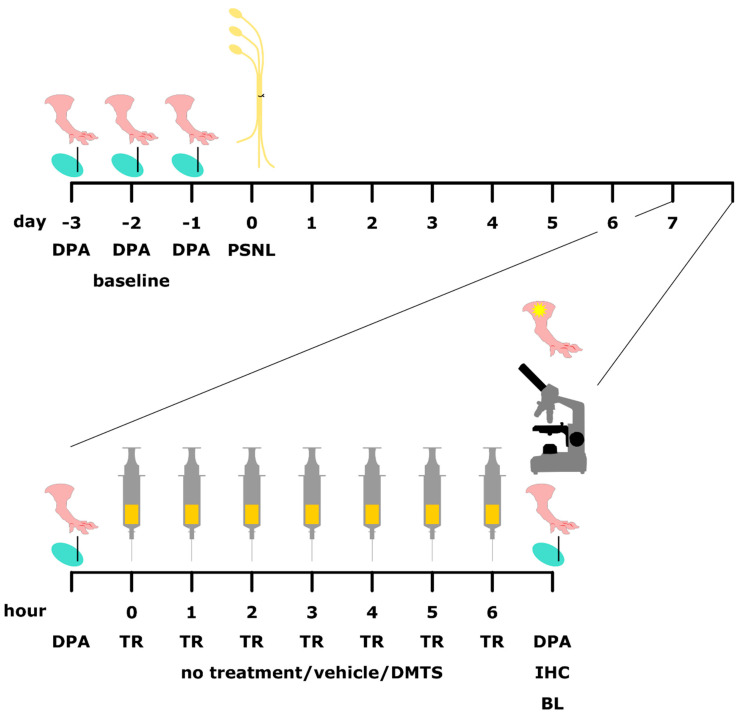
Schematic of the experimental design. DPA: dynamic plantar esthesiometry to detect mechanical pain threshold of hind paws; PSNL: partial sciatic nerve ligation; TR: treatment; IHC: immunohistochemistry of IBA1 in the spinal cord dorsal horn; BL: bioluminescent detection of macrophages at the lesion site.

DMTS solutions were prepared in physiological saline containing dimethyl sulfoxide and polysorbate 80, as described earlier [20]. The final solution included less than 2.5% dimethyl sulfoxide and 0.45% polysorbate 80. Seven days after sciatic nerve surgery, the mechanical pain threshold of mice was checked by dynamic plantar esthesiometry. Thereafter, animals were treated with DMTS (250 µmol/kg, i.p.) or vehicle 7 times in 60 min intervals (Figure 8).

### 4.6. Luminescent Imaging of Macrophages

After performing dynamic plantar esthesiometry on mice with surgically damaged unilateral sciatic nerves, animals were anesthetized with ketamine (120 mg/kg, i.p.) and xylazine (12 mg/kg, i.p.). lucigenin dissolved in PBS (pH 7.4) was injected (25 mg/kg, i.p.). Lucigenin visualizes extracellular superoxide that is produced by NADPH oxidase of macrophages [65]. Luminescent imaging performed 10 min after lucigenin administration (IVIS Lumina II, PerkinElmer). Acquisition time was set to 60 s, F-stop to 1 and Binning to 8 [66]. Luminescent photon flux was expressed as photons/second. Regions of interest were placed over the sites of sciatic nerve injury.

### 4.7. Immunohistochemistry of IBA1

Thirty minutes after the last injection with DMTS or vehicle animals were anesthetized with sodium pentobarbital (80 mg/kg). Transcardial perfusion was initiated with 20 mL PBS (100 mmol/L, pH 7.4) followed by 150 mL of 4% paraformaldehyde. Perfused mice were stored at 4 °C overnight then brains and spinal cords were harvested and stored in 4% paraformaldehyde at 4 °C until processing. Vibratome (Leica VT1000 S, Leica Biosystems, USA) was used to produce 30 µm horizontal sections from brain samples and transversal sections of spinal cords. Sections were stored at −20 °C in sodium phosphate buffer (100 mmol/L, pH 7.4) containing 30% glycerol and 20% ethylene glycol until staining [67]. Sections were washed three times in PBS (pH 7.4). PBS containing 1% hydrogen peroxide was applied for 30 min in order to inhibit endogenous peroxidase activity and washing with PBS was repeated. Slides were treated with citrate buffer (10 mmol/L, pH 6.0) containing 0.05% polysorbate 20 for 10 min at 90 °C followed by washing with PBS after cooling. PBS containing 0.5% Triton™ X-100 was applied for 30 min. Non-specific binding sites were blocked with PBS containing 2% normal goat serum for 30 min. Primary antibody was added in PBS containing 2% normal goat serum (rabbit anti IBA1 antibody 1:10,000, Wako Chemicals GmbH, Germany) [68]. After overnight incubation in a shaker at room temperature, secondary antibody was added for 60 min in PBS containing 1.5% normal goat serum and 0.5% anti-rabbit gamma globulin (Vector Vectastain ABC elite kit PK 6101 anti-rabbit IgG produced in goat). Following washing, 2—2% of A and B solutions of Vectastain^®^ ABC kit were applied in PBS for 60 min. After repeated washing 3,3′-diaminobenzidine (0.02%) was added in Tris buffer (2-Amino-2-(hydroxymethyl)-1,3-propanediol, pH 7.4 with 0.003% hydrogen peroxide and nickel). After being rinsed with PBS, sections were mounted on gelatin-coated slides. Slides were dried overnight at room temperature and dehydrated with increasing concentrations of ethanol and xylene. Slides were covered with DPX mounting medium. Some slides were lost during washing and quality of immunohistochemistry was poor in case of others. This resulted in varying number of slides evaluated per animal (2–13).

### 4.8. Histological Evaluation

Micrographs were produced of the areas of interest using an Olympus IX81 microscope equipped with an Olympus DP74 digital camera. Images were taken in tiff format and were processed with ImageJ software. To count the density of IBA1-positive microglia, 10×-magnification slides were used. Areas of interest were outlined. Images were converted into 8-bit grayscale and grayscale threshold was adjusted until only the cell bodies remained visible. Marked areas were counted within the area of interest by ImageJ. Results were expressed as count of IBA1-positive cells per 100,000 pixel area. Number of IBA1-positive cells was counted in laminae 1–2 of the spinal cord dorsal horn (DH) [69]. To determine activation state of microglia, 20×-magnification images of the same area were utilized. The threshold level automatically offered by ImageJ for the 8-bit grayscale image was accepted and the total area of non-white pixels in the whole image was measured. This area includes both the cell bodies and dendritic processes. The procedure was repeated, but the display threshold was adjusted similarly to that in the densitometry measurement: only cell bodies remained visible. The cell body area was divided by the total cell area providing the activation index. Activation index of activated microglia is closer to 1 because dendritic processes are smaller. Index of resting cells is smaller [70]. Calculation was performed in the whole image. At least three different standard size fields were counted in each area of interest in each slide.

### 4.9. Statistics

Normal distribution of data was checked by Shapiro–Wilk and Kolmogorov–Smirnov tests. In case of data sets that did not represent normal distribution, Kruskal–Wallis test was applied followed by Dunn’s test. One-way ANOVA was preferred for data sets with normal distribution followed by Dunnett’s test or by Tukey’s test. GraphPad Prism 8 software was used for statistical analysis. Data are presented as mean ± standard error of the mean.

## 5. Conclusions

In conclusion, we demonstrate that DMTS alleviates neuropathic pain induced by partial ligation of the sciatic nerve. The effect of DMTS is mediated by TRPA1 and SST_4_ somatostatin receptors. Most probably, SST_4_ receptors of spinothalamic neurons transmit the effect of DMTS-released somatostatin. Inhibition of macrophages at the site of the nerve lesion and that of microglia in the spinal cord dorsal horn can be ruled out in the antineuropathic action of DMTS. Although the precise mechanism of its action has not yet been elucidated, DMTS possesses the potential to become a complementary treatment option for neuropathic pain.

## Data Availability

The data presented in this study are available on request from the corresponding author.
